# Granulocyte colony-stimulating factor (filgrastim) in chemotherapy-induced febrile neutropenia

**DOI:** 10.4103/0971-5851.73590

**Published:** 2010

**Authors:** S. H. Advani, Suvarna Achreckar, Dennis Thomas, Binny Krishnankutty

**Affiliations:** *Department of Oncology, Jaslok Hospital and Research Centre, Mumbai, India*; 1 *Global Medical Affairs, Dr. Reddy’s Laboratories Ltd., Hyderabad, India*

**Keywords:** *Febrile neutropenia*, *filgrastim*, *granulocyte colony-stimulating factor*

## Abstract

**Background:**

The use of granulocyte colony-stimulating factors to treat patients with chemotherapy-induced neutropenia is well accepted. To assess whether administration of filgrastim along with standard empiric antibiotic therapy is beneficial for patients with chemotherapy-induced febrile neutropenia (FN), we conducted an open, non-randomized clinical trial.

**Materials and Methods:**

This was a prospective, open, Phase IV clinical trial in patients receiving chemotherapy for histologically confirmed cancer, with an oral temperature of >38.2°C and absolute neutrophil count (ANC) of <500/mm ^3^. Filgrastim was administered subcutaneously in a dose of 5 mcg/kg/day, 24 hours after administration of cytotoxic therapy, for up to two weeks or until the ANC reached 10,000 cells/mm ^3^. The parameters of assessment included duration of neutropenia, fever, hospitalization and antibiotic usage.

**Results:**

All 24 evaluable patients recovered from neutropenia, fever and FN in a median duration of two days. This result is similar to that reported in earlier studies with filgrastim. Despite the acceleration in recovery from neutropenia and fever, it also reduced the duration of hospital stay and usage of intravenous (IV) antibiotic. Only two adverse events were reported, which were of mild nature.

**Conclusion:**

Filgrastim, when used in patients with chemotherapy-induced neutropenia, exhibited efficacy in accelerating the recovery from neutropenia and fever comparable to that reported with filgrastim in literature. The data from this study suggest that filgrastim is effective in the treatment of chemotherapy-induced neutropenia and is well tolerated by Indian patients.

## INTRODUCTION

Febrile neutropenia (FN) and resultant infections are the major causes of morbidity and mortality in most of the patients receiving chemotherapy. In spite of the standard measures like hospitalization and antibiotics, FN is associated with a significantly high risk of morbidity and mortality in such patients.[1]

The use of dose-intensive chemotherapeutic regimens has made the management of myelosuppression increasingly important. Associated with neutropenia, the occurrence of infections with gram negative organisms is relatively frequent and leads to high morbidity and mortality rates if antibiotic therapy is not begun. Current practice is to initiate prophylactic antibiotic therapy in all the patients who develop FN at the onset of fever. In addition to antibiotic therapy, neutrophil recovery is an important factor for a successful treatment outcome and to avoid treatment delay for the next cycle of chemotherapy in these patients.

Recombinant hematopoietic colony-stimulating factors, which stimulate the production of granulocytes, help to reduce the FN and associated infectious complications of myelotoxic cancer treatment.[2,3]

Granulocyte colony-stimulating factor (G-CSF) has been shown to ameliorate myelosuppression in adults when administered after cancer chemotherapy. G-CSF (filgrastim) has also been used after chemotherapy for myeloid and lymphoid hematologic malignancies in adults, thus showing the feasibility and safety of the drug in this setting. In addition, the period of prolonged neutropenia after myeloablative chemotherapy administered in bone marrow transplantation can be significantly reduced by G-CSF.[4]

Filgrastim (G-CSF) has potent granulopoietic effects that promote the survival, proliferation, differentiation, and function of progenitor and mature neutrophil cells. Filgrastim therapy, started a day after chemotherapy, reduced the severity and duration of neutropenia and decreased by nearly half the incidence of FN and the duration of hospitalization and antibiotic usage with associated pharmacoeconomic benefits to patients. The use of filgrastim to prevent episodes of FN in patients with cancer who receive chemotherapy is now approved in many countries.[3]

Against this background, a clinical trial was undertaken to assess the efficacy of G-CSF (filgrastim), judged primarily by the mean number of days required to restore the depressed absolute neutrophil count (ANC) and also the tolerability of the regimen.

## MATERIALS AND METHODS

This prospective and open labeled study was conducted at Jaslok Hospital and Research Centre, Mumbai, from April 2005 to November 2006, after obtaining necessary approval from the institutional ethics committee (IEC).

The study included 29 adult patients receiving chemotherapy for histologically confirmed cancer, who had an oral temperature >38.2°C and ANC level <500/mm^3^. All the patients who were coming under World Health Organization (WHO) performance scale 0–2 and willing to give informed consent were recruited.

Filgrastim was administered subcutaneously in a dose of 5 mcg/kg/day, 24 hours after administration of cytotoxic therapy, for up to two weeks or until the ANC reaches 10,000 cells/mm^3^. After the administration of filgrastim, the ANC level and oral temperature were measured and documented every six hours. The details of antibiotics used and number of days of hospitalization were also captured.

Recovery from neutropenia, fever and FN was considered as the primary efficacy variable and duration (in days) of intravenous antibiotic usage and hospitalization were the secondary efficacy variables.

Investigations for safety and tolerability were done before and after the administration of filgrastim. All clinically significant adverse events reported were documented.

### Definitions

Number of days of neutropenia: The number of days the ANC count remained less than 500/mm^3^.

Number of days of fever: The number of consecutive days of fever (including the day of presentation) when the oral temperature was 37.5°C or higher, plus any subsequent days when the temperature was >38.2°C and ensuing days with peaks of 37.5°C.

Number of days of FN: The number of days required to achieve both a temperature less than 37.5°C and ANC of 500/mm^3^ or more.

Thrombocytopenia:[5] Platelet count less than 50,000 mm^3^.

### Immunogenicity

Serum was collected for antibody screening at baseline and at the end of the cycle. Serum samples were analyzed to detect the presence of specific IgG antibodies to G-CSF by using enzyme-linked immunosorbent assay (ELISA) method.

### Statistical analysis

Per protocol analysis was carried out for both efficacy and safety variables. Patients with missing values were not taken for that particular analysis. STATA version 10.1 (Stata Corp, College Station, TX) was used for statistical analysis. The efficacy variables that included duration of neutropenia, ANC level, duration of FN, number of days of fever, duration of IV antibiotic usage were analyzed using descriptive statistics. All the biochemical parameters were evaluated before and after the administration of G-CSF. These values were analyzed using paired t-test and McNemar’s test.

### Ethics

The study was conducted in accordance with Good Clinical Practices and the Declaration of Helsinki as amended in Edinburgh, Scotland (October 2000). The study protocol and statement of informed consent were approved by the IEC prior to study initiation. Written informed consent was obtained from each patient prior to entry into the study, in compliance with regulatory requirement.

## RESULTS

A total of 29 patients were enrolled in the study and 28 patients received filgrastim therapy along with radiation. Of the 28 evaluable patients, four were not taken for primary efficacy analysis as the initial neutrophil counts in two patients were more than 500/mm^3^ and two dropped out before achieving target neutrophil level. The demographic characteristics of patients are summarized in Table 1. There were more male patients (67.9%, n=19) than female patients (32.1%, n=9).

**Table 1:** Demograph

Characteristics	Mean (n=28)	SD	Median	Range
Age (years)	49.71	16	52.50	20–75
Height (cm)	161.39	9.91	159.5	143–185
Body weight (kg)	62.36	9.07	60	45–89
BSA (m^2^)	1.66	0.14	1.64	1.36–2.15

n, number of patients; BSA, body surface area; SD, standard deviation

### Neutropenia and fever

**Figure 1:**
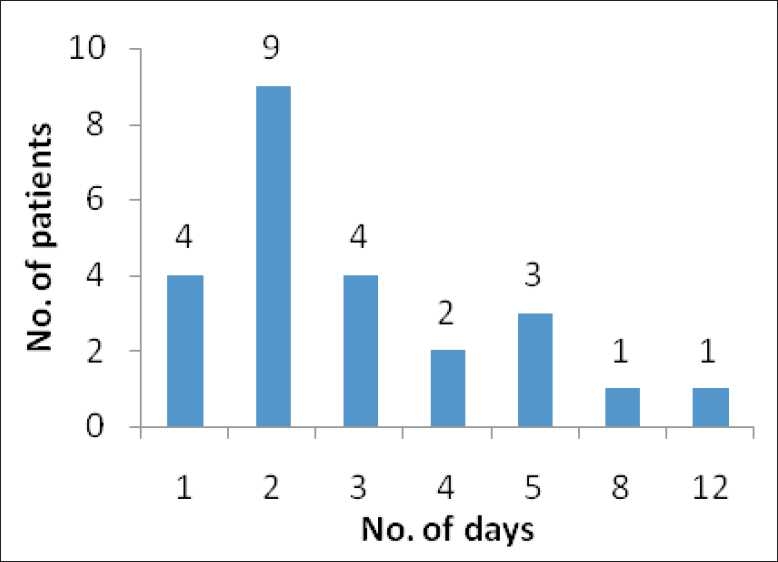
Resolution of neutropenia: number of patients recovered from neutropenia in each particular day

Neutropenia: It was found that neutropenia resolved in all the 24 patients within 12 days of filgrastim therapy (range 1–12 days). The median time was two days. The number of patients who recovered from neutropenia on each day is presented in Figure 1. Four patients resolved from neutropenia on the first day of filgrastim therapy. Nine patients recovered on the second day. Only for two patients, the ANC level remained <500 cells/mm^3^ after five days of therapy. All the patients recovered within 12 days.

Fever and FN: All patients became afebrile and the median time for resolution of both fever and FN was two days. Fever resolved in all patients within 14 days of therapy (range 1–14 days) and FN resolved within 12 days of therapy (range 1–12 days). The primary efficacy results are presented in Table 2.

**Table 2:** Primary efficacy parameters

Parameter	No. of patients	Days (mean±SD)	Median	95% CI of mean	Range
Neutropenia (ANC <500 cells/mm^3^)	24	3.21±2.50	2	(3.18, 3.24)	(1,12)
Fever	24	3.17±3.02	2	(3.13, 3.21)	(1,14)
FN	24	2.33±2.51	2	(2.30, 2.37)	(1,12)

ANC, absolute neutrophil count; SD, standard deviation; CI, confidence interval; FN, febrile neutropenia

### Hospitalization and IV antibiotic usage

The duration of hospitalization ranged between 1 and 25 days, with a median of five days. The maximum number of days of IV antibiotic usage was 15 days, with a median duration of four days. The secondary efficacy results are presented in Table 3.

**Table 3:** Secondary efficacy parameters

Parameter	No. of patients*	Days (mean±SD)	Median (days)	95%CI of mean	Range
Hospitalization	27	6.81±5.68	5	(6.75, 6.88)	(1,25)
IV antibiotic usage	26	5.08±3.05	4	(5.04, 5.11)	(2,15)

IV, intravenous; SD, standard deviation; CI, confidence interval

* One patient’s data for hospitalization and two patients’ data for IV antibiotic usage were missing

### Safety analysis

Two adverse events (myalgia, vomiting) were reported in two patients during the study. Both were mild in nature. Myalgia could possibly be due to the study drug and vomiting was definitely not due to the study drug. There were no serious adverse events reported in this study.

There were no statistically or clinically significant alterations in serum creatinine, aspartate transaminase (AST), alanine transaminase (ALT) or hemoglobin levels after treatment with G-CSF compared to baseline.

### Thrombocytopenia

Of the eight patients who had thrombocytopenia at baseline, six patients recovered at the end of cycle while two patients continued to have thrombocytopenia. Of the 16 patients who did not have thrombocytopenia at baseline, two had thrombocytopenia at the end of treatment.

### Immunogenicity

There was no evidence of formation of neutralizing antibodies. It was found that there was no significant difference in G-CSF antibody levels between baseline and the end of cycle (*P*>0.05).

## DISCUSSION

In this study, the use of filgrastim in patients undergoing chemotherapy resulted in a desirable clinical response. Various studies had been carried out earlier to assess the performance of filgrastim when used in patients undergoing chemotherapy. All these studies concluded that the use of filgrastim accelerated the neutrophil recovery and shortened the duration of fever, FN and hospitalization in patients with cancer who had received chemotherapy.[2–8] These studies are the sources of historical data of filgrastim, published in literature.

The median duration of neutropenia after filgrastim administration was two days. This result is similar to that reported in a study by Hartmann *et al*.[2] However, in another study with filgrastim, Masher *et al*. reported a median duration of neutropenia of three days,[3] which is slightly longer than that observed in the present study. Likewise, the performance of filgrastim in reducing the duration of fever (two days) was also better in our study when compared to the data of three and seven days reported by Masher*et al*.[3] and Heil *et al*.,[5] respectively. The median time for the resolution of FN was also shorter when compared with the results of earlier studies on filgrastim.[3,4] Thus, filgrastim’s efficacy in reducing duration of neutropenia, fever and FN in this study was either similar to or better compared to that reported in earlier studies.

The median duration of hospitalization was five days, whereas Masher *et al*. reported that the median duration of hospitalization was eight days.[3] The median number of days of IV antibiotic usage was four days compared to 18.2 days reported by Welte *et al*. in a Phase III study.[4] In this study, filgrastim was found to be well tolerated.

A reduction in the duration of hospitalization and IV antibiotic usage can significantly reduce the treatment cost. An accurate assessment of cost-benefit could not be made as our study was not designed for such pharmacoeconomic analysis. However, the benefit of reduced cost of hospitalization and IV antibiotics should be considered when the filgrastim treatment is given.

## CONCLUSION

Filgrastim, when used in patients with chemotherapy-induced neutropenia, exhibited efficacy in accelerating the recovery of neutropenia and fever comparable to that reported with filgrastim in literature. The data from this study suggest that filgrastim is effective in the treatment of chemotherapy-induced neutropenia and is well tolerated by Indian patients.

## References

[1] Harousseau JL, Witz B, Lioure B, Hunault-Berger M, Desablens B, Delain M (2000). Granulocyte colony-stimulating factor after intensive consolidation chemotherapy in acute myeloid leukemia: Results of a randomized trial of the group oust-est leucemies aigues myeloblastiques. J Clin Oncol.

[2] Hartmann LC, Tschetter LK, Habermann TM, Ebbert LP, Johnson PS, Mailliard JA (1997). Granulocyte colony-stimulating factor in severe chemotherapy induced afebrile neutropenia. N Engl J Med.

[3] Maher DW, Lieschke GJ, Green M, Bishop J, Stuart-Harris R, Wolf M (1994). Filgrastim in patients with chemotherapy-induced febrile neutropenia: A double-blind, placebo-controlled trial. Ann Intern Med.

[4] Welte K, Reiter A, Mempel K, Pfetsch M, Schwab G, Schrappe M (1996). A randomized phase-111 study of the efficacy of granulocyte colony-stimulating factor in children with high-risk acute lymphoblastic leukemia. Berlin-Frankfurt-Münster Study Group. Blood.

[5] Heil G, Hoelzer D, Sanz MA, Lechner K, Liu Yin JA, Papa G (1997). A randomized, double-blind, placebo-controlled, phase III study of filgrastim in remission induction and consolidation therapy for adults with de novo acute myeloid leukemia. The International Acute Myeloid Leukemia Study Group. Blood.

[6] Neupogen (Filgrastim) http://www.neupogen.com/pdf/Neupogen_PI.pdf.

[7] Larson RA, Dodge RK, Linker CA, Stone RM, Powell BL, Lee EJ (1998). A randomized controlled trial of filgrastim during remission induction and consolidation chemotherapy for adults with acute lymphoblastic leukemia: CALGB study 9111. Blood.

[8] Ghalaut PS, Sen R, Dixit G (2008). Role of granulocyte colony stimulating factor (G-CSF) in chemotherapy induced neutropenia. J Assoc Physicians India.

